# Does the macro design of an implant affect the accuracy of template-guided implantation? A prospective clinical study

**DOI:** 10.1186/s40729-021-00320-3

**Published:** 2021-04-26

**Authors:** Sigmar Schnutenhaus, Cornelia Edelmann, Heike Rudolph

**Affiliations:** 1 Zentrum für Zahnmedizin Dr. Schnutenhaus MVZ GmbH [Center for Dentistry, Dr. Schnutenhaus Community Health Center (CHC) GmbH], Breiter Wasmen 10, 78247 Hilzingen, Germany; 2 Clinic for Dental Prosthetics, Center for Dental, Oral and Maxillofacial, Ulm University, Albert-Einstein-Allee 11, 89081 Ulm, Germany

**Keywords:** Dental implant, CBCT, Computer-guided surgery, Accuracy, Sleeve, Computer-assisted surgery, Implant design

## Abstract

**Background:**

An implant prosthesis aims to ensure the best possible rehabilitation of function and esthetics following tooth loss. Template-guided insertion is used to achieve an optimal position of the implant with regard to prosthetic restorability, bone availability, and condition of the surrounding soft tissues. The accuracy of template-guided implant placement is subject to various influencing factors.

The clinically achievable accuracy depending on the macro design of the implant body was investigated in this prospective clinical study.

**Material and methods:**

In this prospective clinical study, 20 implants were placed in 20 patients. The implant had a pronounced conical outer geometry (Conelog ProgressiveLine, Camlog Wimsheim, Germany). Data from a study using an implant with a distinct cylindrical outer geometry were used as a comparison group (Conelog ScrewLine, Camlog, Wimsheim, Germany). The clinically achieved implant position was compared with the planned position.

**Results:**

The evaluation of the two-dimensional deviations in direction resulted in the following mean values (standard deviation) at the shoulder: 0.42 mm (0.33) in the buccolingual direction, 0.27 mm (0.25) in the mesiodistal direction, and 0.68 mm (0.55) in the apicocoronal direction. The mean angular deviation was 4.1° (2.3). The three-dimensional (3D) deviation was 0.94 mm (0.53) at the shoulder and 1.36 mm (0.62) at the apex of the implant.

Significant differences between implants with different macro designs were found in the apicocoronal direction. In connection to this, a significant 3D deviation was found at the implant shoulder.

**Conclusions:**

Significant differences in height were found between the groups. The study had shown that the macro design of an implant has no influence on accuracy in all other directions. Overall, the implants showed a high level of accuracy and a low variation in values. The values were in the range determined by the template-guided insertion system in numerous other investigations. This provides good predictability of prosthetic rehabilitation.

**Trial registration:**

German Register for Clinical Studies (DRKS-ID: DRKS000018939). Date of registration: November 11, 2019.

## Background

The objective of implant-prosthetic restoration is to replace the lost teeth with a predictable and durable restoration in terms of function, as well as esthetics. Three-dimensional (3D) planning opens up possibilities to determine the optimal position and number of implants. For a successful implant prosthesis, it is crucial that the prosthetic goal be defined in terms of function and esthetics, on the basis of this 3D plan. Anatomically adjacent structures should be considered in 3D planning [1], which could then be implemented using computer-assisted surgical procedures.

Template-guided implant placement has proven to be a reliable static computer-assisted surgical method. A further dynamic procedure is real-time navigation [2, 3].

The proven procedure of conventional freehand implantation clearly shows greater inaccuracies during implant insertion compared to template-guided implantation [4–7]. Tahmaseb et al. reviewed 20 clinical studies regarding the accuracy of statically navigated implant placement. The mean deviation was found to be 1.2 mm at the implant shoulder and 1.4 mm at the implant tip, while the mean angular deviation was 3.5° [8].

Significant differences in accuracy were found when comparing fully guided and pilot drill-guided computer-assisted implantations. Fully guided procedure showed significantly more accurate results with respect to clinically relevant parameters, such as angular deviation, deviations at the implant shoulder, and the apex [9, 10].

In a randomized clinical trial, Varda et al. compared the deviations resulting from freehand implantation and computer-assisted implantation procedures using drilling templates. For implantations with sleeve-guided drilling templates, an average angular deviation of 3.04° with a range of 0.4°–6.3° was observed, whereas in a freehand procedure, the mean value reached 7.03° ranging between 0.7°–21.3° [9]. Large deviations with respect to 3D position and angle have a direct influence on the prosthetic sustainability of the implants.

Numerous studies on guided implant placements have attempted to determine factors that affect the accuracy of the procedure. The manufacturing process for the drilling template can have an impact on the accuracy of the implant placement. Several studies have reported the influence of additive printing and subtractive processes, as well as of the materials used in these processes [11–13].

The surgeon’s experience could have a possible influence on the accuracy achieved in a procedure. However, the use of static computer-assisted surgery appears to be only slightly dependent on the surgeon’s experience [14]. A learning curve that leads to better results when preparing the implant bed could not be demonstrated in a clinical study [15]. However, it can be shown that the experience of the user in positioning the template has a significant influence on the accuracy [16]. Several studies have discussed the influence of the drill sleeve on accuracy [17–19]; the design of the drill sleeve was found to be a cofactor in accuracy.

The support of the template demonstrates a significant effect on the accuracy, as evidenced in clinical studies wherein bone-supported, mucosa-supported, and tooth-supported guides have resulted in significantly different outcomes [20]. For example, mucosa-supported templates show a significantly lower precision when compared to bone- or tooth-supported templates [20, 21].

The surgical approach may also have an impact on the precision of implant placement. In a fully guided procedure with a flapless approach, significantly more accurate implant positions could be achieved compared to open flap procedures [22]. On the other hand, in semi-guided procedures, it has been shown that implants placed under direct visual control achieve a more precise position [23].

Various anatomical features have been cited as possible factors affecting accuracy. Bone hardness has a marked influence on the correlation between planned and achieved implant positions [24, 25]. A clinical study demonstrated that the type and size of the edentulous jaw areas had no influence on accuracy [26]. Several studies have assessed whether implant insertion in the upper or lower jaw is a significant factor [4, 22, 26].

Few studies have directly compared different implant systems in guided implant placement. An in vitro study showed that the accuracy depends on the implant system [27]. Clinical experience shows that deviations in direction can be deliberately performed during implant placement, particularly with powerful self-tapping implant systems. It can thus be assumed that the macro design of the implant can also influence the achieved accuracy.

In summary, template-guided implantation appears to be largely independent of the anatomical situation, whereas the procedures and technical aspects of implantation have a clear influence.

The objective of this prospective clinical study was to determine the dependent factors of the accuracy of template-guided implant placement after 3D planning. The aim of this study was to clarify whether the macro design of an implant has an influence on accuracy.

## Material and methods

### Patients

Twenty patients were included in this prospective clinical study. Patients were recruited following registration of the study with the German Clinical Trials Register (DRKS-ID: DRKS000018939) and approval by the responsible ethics committee of the State Medical Association of Baden-Württemberg, Germany (Application No.: F2019-057-z). Implants were placed during the period from February 2020 to July 2020. Patients’ data were collected in the practice of PD Dr. Schnutenhaus in Hilzingen (cooperating partner of the Clinic for Dental Prosthetics, Ulm University Hospital). In cases where multiple implants were to be placed in a patient, a test implant was specified to maintain independent values. After planning the position and number of implants, the test implant was determined preoperatively by randomization using the randomization function in Excel (Microsoft Corporation, Redmond, WA, USA).

The following criteria were formulated for inclusion in the study:

Inclusion criteria:

- Submission of written informed consent
- Restoration of at least one missing tooth using an implant
- At least five residual teeth in the affected jaw

Exclusion criteria:

- People under 18 years or people without legal capacity
- Use of a template for implant placement is not possible (restricted mouth opening)
- Necessary additional augmentation requirements
- Heavy smoker (> 10 cigarettes/day)
- Immediate implant placements
- Intake of bisphosphonates
- Pregnant women
- Alcohol and/or drug abuse
- Patients with infectious diseases, such as hepatitis, HIV, or AIDS
- Poorly controlled diabetes mellitus

### Planning

After obtaining written informed consent from the patients, cone beam computed tomography (CBCT) was performed (Gendex CB500, Gendex Dental Systems. Des Plaines, USA). The image was acquired with a constant 0.2 voxel resolution. For implant planning, an alginate impression of the concerned jaw was made to fabricate a diagnostic plaster model for each patient followed by a prosthetic wax-up, which was then optically scanned (3Shape Scanner D 700 3Shape A/S, Copenhagen, Denmark). The 3D implant planning was performed with the implant planning software SMOP (Swissmeda AG, Baar, Switzerland). This made it possible to overlay the CBCT data with the standard tessellation language (STL) data sets of the patient models using the corresponding program function of the planning software. The optimal implant position was determined based on the information regarding the availability of bone, planned prosthetic restoration, and condition of the soft tissues. This implant planning was then saved as an interface data record. Using this plan, the optimal implant position was defined. All planning steps and the subsequent implant placement were performed by the same practitioner (SiS). Based on this plan, a drilling template was designed by an external service center (Camlog Dedicam, Wimsheim, Germany). The design data of the drilling templates were then sent to the dental laboratory in the clinic. For the fabrication of the drilling template, it was stipulated that tooth-support had to be carried out. All drilling templates were fabricated by the same dental technician using a 3D printer (Version 3, Formlabs Inc., Somerville, MA, USA). The templates were cleaned and post-cured according to the manufacturer’s instructions. After inserting the respective drill sleeves, the templates were sterilized in the steam sterilizer and made available to the surgeon in a shrink-wrapped condition.

### Implantation

All implants were placed by an experienced surgeon (SiS). Surgical procedures were performed with local anesthesia. After elevating the mucoperiosteal flap, the implant bed was prepared according to the manufacturer’s protocol. To reduce other influencing factors, the procedure was completely template-guided, including the insertion of implant.

### Registration of the implant position

All implants were provided with fixed dentures. For prosthetic restoration, the clinical situation was recorded three months (± 2 weeks) post-implantation using an individual tray, impression post, and addition silicone impression material (imprint Quick, 3 M Espe, Seefeld, Germany). All impressions were made by one operator (SiS). After disinfection, the impression was transferred to a plaster model by a dental technician. The impression post was then supplemented with a screwed-on analogous implant and the impression was digitized (3Shape Scanner D 700, 3Shape A/S, Copenhagen, Denmark).

Overlay data sets were created using the Geomagic Studio program (Version 9, Geomagic, NC, USA). All data were consecutively analyzed in terms of location and time by an investigator, regardless of their generation. The data records of the digitized implant impressions were exported as interface files in STL format. The latter represented the clinically achieved implant position. The 3D interface dataset of the implant planning exported from the planning program (SMOP) served as the reference data set.

Data sets were reduced to a defined structure, the unchangeable hard tooth substance, in order to exclude errors due to soft tissue changes or deviating implant positions.

For the planned analysis of the distance and angle deviations, the use of auxiliary constructs was necessary, which reflected the exact planned position of the implant and the clinically achieved implant position. It was created with the aid of the Surfacer 10.6 program (Imageware, Ann Arbor, MI, USA) using simple geometric shapes. The auxiliary constructs were adapted to the respective implant lengths and diameters and then loaded into the Geomagic Studio program for assignment. Thus, it could be ensured that the axis end points and the axis deviation of the implant positions could be determined in a standardized manner for further analysis. This methodology has already been extensively used and described by Schnutenhaus et al. [28].

The assigned auxiliary constructs, which reproduced the key data of the 3D information of the planned and clinically achieved implant position, were loaded into the Surfacer 10.6 Imageware program for further analysis.

### Analysis of the implant position

**Fig. 1:**
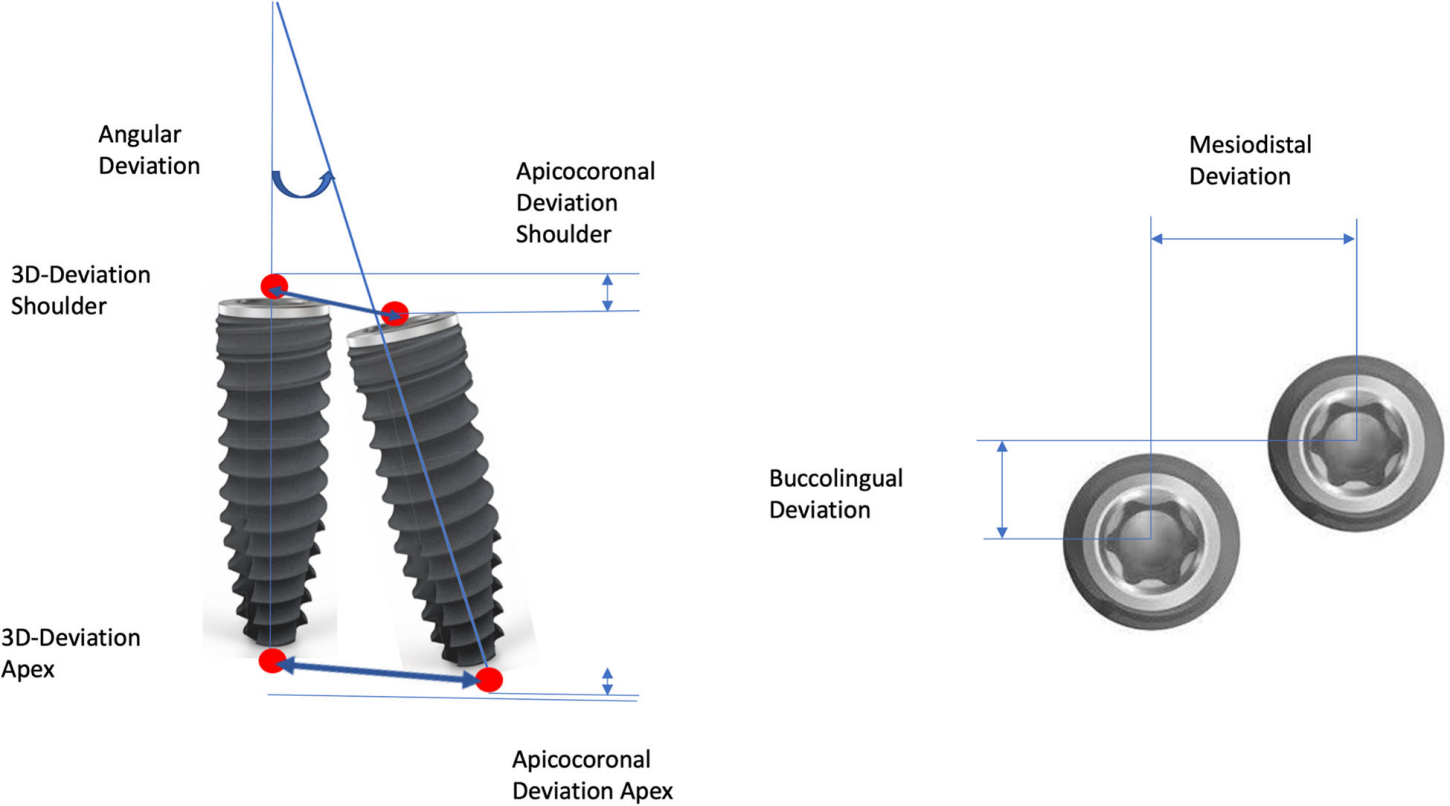
Representation of the measuring distances and the angular deviation between the planned and actually achieved implant positions

The metric analysis included the following measurements (Fig. 1):

- Three-dimensional deviation: the 3D deviation of the midpoints between the planned implant position and clinically achieved implant position, measured at the implant shoulder and apex (corresponding to the Euclidean distance).
- Apicocoronal deviation (height difference): vertical spatial offset measured at the center of the implant shoulder.
- Axis deviation: angular deviation of the implant axes from planned and clinically achieved implant position.
- Two-dimensional (2D) deviation in the mesiodistal and buccolingual directions measured at the implant shoulder and axis.

The measurement method was based on the principle of Tahmaseb et al. [29] to enable better comparability with current and future studies.

### Comparison group

**Fig. 2:**
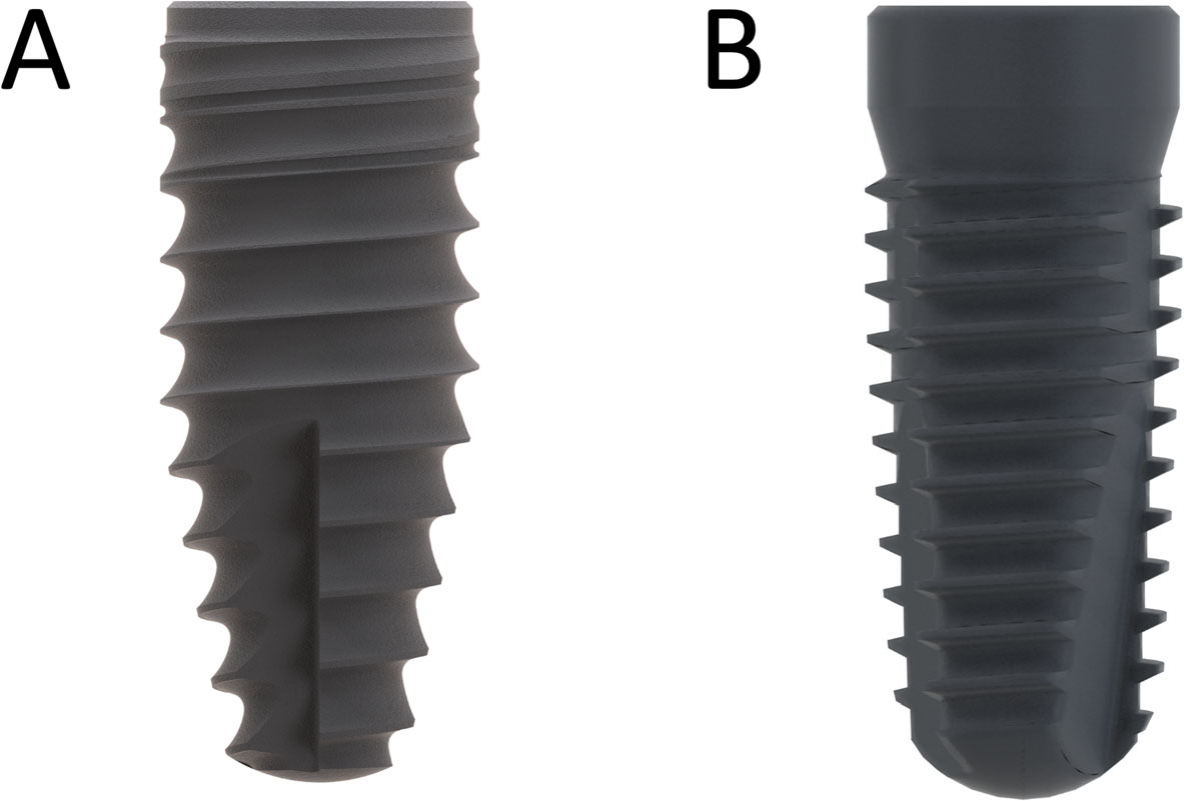
External geometry of the more conical test implant (**a**) and the more cylindrical comparison implant (**b**)

The test implant (Conelog ProgressiveLine, Camlog Wimsheim, Germany) had a pronounced conical outer geometry. The present study compared the influence of the external geometry with a more cylindrical implant body (Conelog ScrewLine, Camlog, Wimsheim, Germany) (Fig. 2).

The data of the comparison group was extracted from a previous study [25].

### Power calculation

Since there was no available data from studies on the influence of different macro designs of implants on accuracy, the power analysis was performed from our own data on the influence of different drilling templates, to estimate the sample size [28, 30]. The minimum required sample size of 9–15 implants according to the platform, apex, and angle deviation was calculated using a statistical software (G*Power software version 3.1. Erdfelder, Faul & Buchner, 1986) for the Mann-Whitney *U* test with 80% of study power and a significance level (*α*) of 0.05.

### Statistical analysis

The mean values, standard deviations, 95% confidence intervals, and the minimum and maximum values were provided for the variables. After testing for normal distribution, statistical testing was performed. For values that were normally distributed, a *t* test was performed for the mean values in independent random samples to compare the planned and achieved implant positions. If the values were not normally distributed, the Mann-Whitney *U* test was performed.

A *p* value of < 0.05 was considered statistically significant. Statistical analysis was performed using IBM SPSS® Statistics Version 26.

## Results

### Description of the study population

Twenty patients were included in the test group. The comparison group comprised of 48 patients. The demographic data as well as information on the implant position and the implants used are shown in Table 1. With strict adherence to the protocol, all 20 included patients were operated without complications. The evaluation of all 20 patients is shown in Table 2. No 2D deviations were observed in the comparison group. Group comparison was performed using Mann-Whitney *U* test, as there was no normal distribution.

**Table 1:** Demographics of the patients and distribution of the implants

	Test group (*N* = 20)	Comparison group^a^ (*N* = 48)
Patient age (years)		
Average age	60.8	52.2
Min–max	30–86	24–77
Gender		
Female	12	31
Male	8	17
Type of arch		
Upper jaw	8	48
Lower jaw	12	0
Implant location		
Anterior	2	19
Premolar	5	28
Molar	13	7
Implant diameter (mm)		
3.3		1
3.8	7	32
4.3	13	15
Implant length (mm)		
7.0		1
9.0	8	8
11.0	11	20
13.0	12	19

^a^Schnutenhaus et al. Alveolar ridge preservation and primary stability as influencing factors the transfer accuracy of static guided implant placement: a prospective clinical trial. BMC Oral Health, 2020; 20; 178 [25]

**Table 2:** Deviations between the planned and actually achieved implant positions

	Test group *N* = 20			Comparison group *N* = 48			Group comparison *p value*
	Mean (SD)	95% CI	Min–max	Mean (SD)	95% CI	Min–max	
Deviation at implant shoulder (mm)							
3D	0.94 (0.53)	0.69–1.19	0.15–2.15	0.66 (0.30)	0.58–0.75	0.18–1.66	**0.024**^a^
Mesiodistal	0.27 (0.25)	0.15–0.39	0.04–1.00				
Buccolingual	0.42 (0.33)	0.26–0.57	0.01–1.11				
Apicocoronal	0.68 (0.55)	0.43–0.95	0.01–1.92	0.28 (0.27)	0.20–0.36)	0.00–1.40	**0.002**^a^
Deviation at implant apex (mm)							
3D	1.36 (0.62)	1.05–1.64	0.31–2.92	1.36 (0.65)	1.17–1.55	0.26–3.50	0.682
Mesiodistal	0.67 (0.53)	0.43–0.92	0.07–2.00				
Buccolingual	0.74 (0.56)	0.47–1.00	0.06–1.87				
Apicocoronal	0.67 (0.51)	0.43–0.91	0.10–2.06				
Angular deviation (degree)	4.1 (2.3)	3.0–5.2	0.4–10.0	4.0 (2.1)	3.4–4.6	0.4–11.1	0.916

Group comparison between the test and comparison group using the Mann-Whitney *U* test with a *p* value < 0.05 (bold with ^a^)

**Fig. 3:**
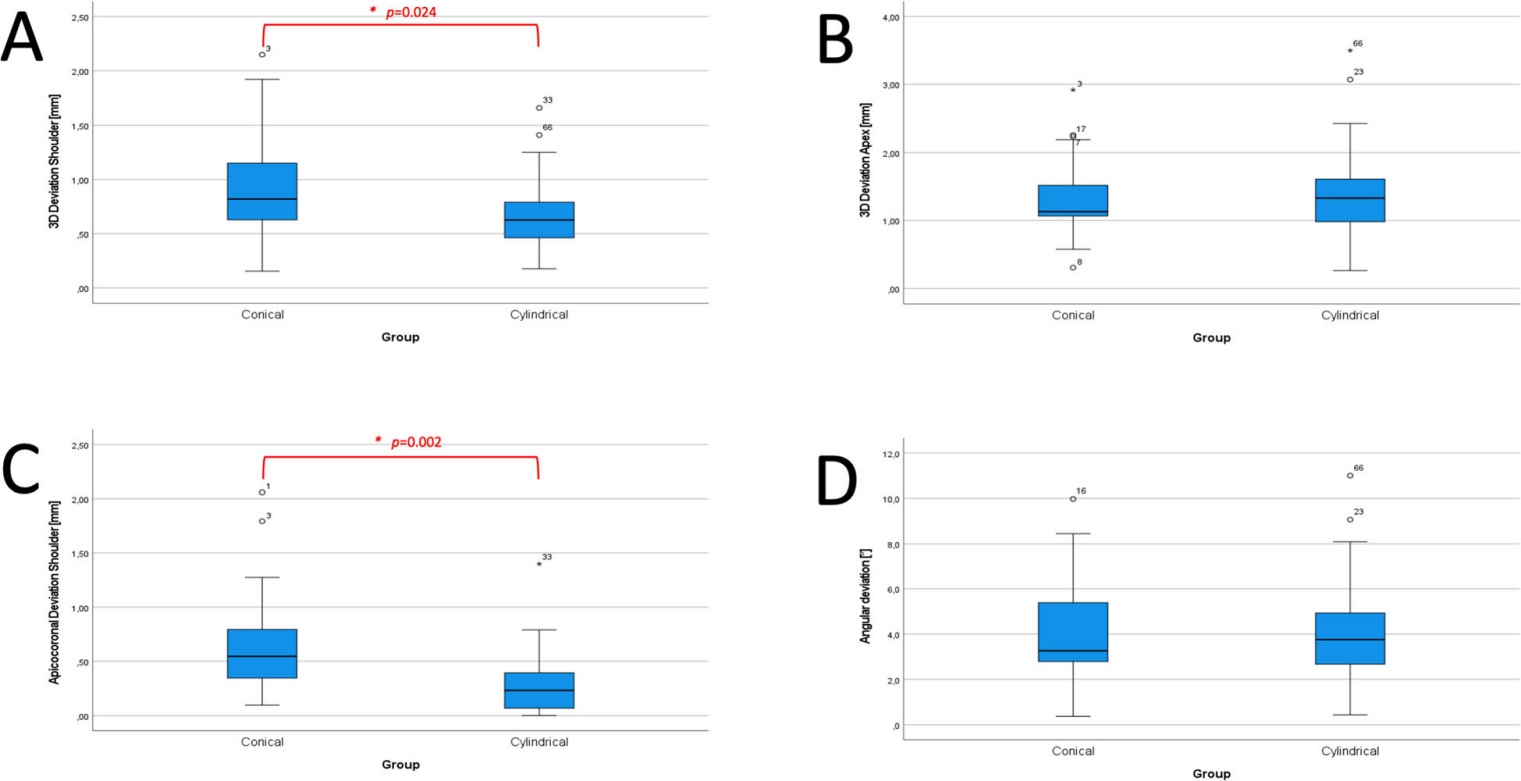
Significant differences (*) observed between the groups with respect to the 3D deviations (**a**) and the apicocoronal distance (**c**) at the implant shoulder. There were no significant differences in the measurement of 3D deviation at the apex (**b**) and in angular deviation (**d**)

The evaluation of implants in their respective groups showed significant deviations in the apicocoronal direction with associated 3D deviation on the shoulder (Fig. 3). There were no significant differences in angular deviation

The evaluation of the 2D deviations in the mesiodistal and buccolingual directions of the test group resulted in mean values of 0.27 mm and 0.42 mm, respectively at the shoulder, and 0.67 mm and 0.74 mm, respectively at the apex. The deviations in the mesiodistal and buccolingual directions were not significantly different.

The subsequent calculation of the sample size resulted in a necessary implant count of 136 implants for 3D deviation in the apicocoronal direction. No sample size could be calculated for 3D deviation at the apex and angular deviation since the effect size was < 0.00001.

## Discussion

As an option for computer-assisted surgery, template-guided implant placement is an established procedure. The existing literature shows that template-guided implant procedure is superior to free hand implant placement. In a clinical study by Vercruysen et al., various template-guided procedures were examined in comparison to the freehand method [7]. With the freehand implant procedure, significantly higher deviations were found averaging 2.77 mm (range 0.33–8.34 mm) at the implant shoulder, 2.91 mm (range 0.53–7.37 mm) at the apex, and 9.92° (range 1.45°–27.76°) for angular deviation. Another clinical study by Varga et al. observed a mean deviation of 1.82 mm (range 0.56–5.38 mm) at the implant shoulder, 2.43 mm (range 0.54–4.83 mm) at the apex, and an angular deviation of 7.03° (range 0.71°–21.30°) in freehand implant bed preparations [9]. Further comparative clinical studies on freehand and computer-assisted procedures consistently showed significant differences in all parameters of accuracy and a large range between the minimum and maximum values [5, 9, 31, 32]. In particular, a higher number of deviations in the angular dimensions were found in the clinical studies. For example, Aydemir and Arisan observed a mean deviation of 10.04° with a range of 2.19–20.42° when using the freehand method [33].

The transfer of virtually planned implants to the real situation is possible with template-guided procedures with a high degree of accuracy from a clinical point of view. This accuracy cannot be achieved with partially guided insertions (e.g., guided pilot drilling) and freehand implant placements [34]. In a systematic review by Tahmaseb et al., a mean deviation of 1.2 mm at the implant exit point, 1.4 mm at the apex, and a mean angular deviation of 3.5° were reported [8]. Similar values can be found in the systematic review and meta-analysis by Bover-Ramos et al. [35]. They observed values of 1.08 mm at the shoulder, 1.35 mm at the apex, and an angular deviation of 3.62°. The respective accuracies achieved in the present study fit into this order of magnitude. Numerous other studies have shown similar results. Thus, it can be stated that precision is inherently limited in the system. The surgeon must bear this in mind when planning for implant placement [36]. The mean deviations achieved at the implant exit point and angle influence the predictability of prosthetic restoration.

The values that can be achieved with a template-guided procedure are considerably more precise and, above all, show a lower scattering of the measurement values. Even if such good accuracy can be achieved in the clinical context with the values showing little variation between the individual systems, it is important to consider other influencing factors.

The manufacturing process for the drilling template can have an impact on the accuracy of the implant placement. As observed in several in vitro studies, templates from different 3D printers led to significant differences in the accuracy of the placed implants [11, 37]. However, another study using templates from different printers did not demonstrate any significant differences [38]. In a study on the influence of two different printing materials for templates, no significant differences were found [13]. An in vitro study by Henprasert et al. showed that the fabrication of the template in an additive or subtractive process had no influence on the implant position [12]. In contrast, Abduo and Lau demonstrated that templates produced through subtractive manufacturing led to more precise results [39]. The template design also influences the accuracy of implant position; in a clinical study, significantly lower deviations in the implant positions were measured in a system with two guide sleeves compared to the known mean values from the reviews [30].

Putra et al. showed that bone density has a significant influence on accuracy. With lower bone density, a higher deviation was observed in the implant positions [24]. This finding can be corroborated by another study which demonstrated that reduction in primary stability resulted in lower accuracy [25]. Regarding the position of the edentulous space in different configurations of residual dentition, the comparison of the free-end situation with gaps in teeth showed no influence on the accuracy [26]. In an in vitro study by Abdou and Lau, implant location exhibited no influence on the accuracy of fully guided protocol. There was no decrease in the achieved accuracy, whether it was an anterior implant or posterior implant [40].

The type of drill sleeve has an influence on the accuracy of the implant position. A normal template with a guide sleeve height of 5 mm or less can introduce large deviations in implantation, resulting in significant levels of inaccuracy [17]. Tallarico et al. showed that open sleeves resulted in poor accuracy compared to closed sleeves. It could also be determined that conventional templates with metallic sleeves produced less precise results than surgical templates that only had the guide in the 3D printed template material [18]. Only few studies have investigated the implant system has only been investigated in a few studies as a cofactor for precision. For example, Yeung et al. found significant angular deviations and vertical deviations in an in vitro study using three different implant systems [27]. In particular, high and clinically relevant deviations in the vertical dimension were determined in this study. This is in line with the results of the clinical study presented here.

The present study shows that macro design can influence the accuracy of implant placement. Based on clinical observations, this can be interpreted through different preparation methods of the implant bed. It turns out that the drilling protocol is dependent on the implant system. During the preparation of the osteotomy of the test implant, an additional preparation was performed with a dense bone drill or thread cutter.

With this finding, a learning curve of the user for each implant system is to be expected again, which will lead to an increased accuracy in the vertical offset. An adaptation of the drilling protocol or drill sequences can also result in increased accuracy in the apicocoronal direction. Clinical investigations on the effect of different implant systems on accuracy appear to make sense from the present investigation. However, these studies on the factors of accuracy are still underrepresented. The present research is intended as a contribution to these studies.

## Conclusions

Template-guided implant placement is a proven procedure. The present study shows that there are only minor deviations between the planned and the achieved implant positions for implants with significantly different macro designs. Significant differences were found pertaining to deviation in height. The study had shown that the macro design of an implant has no influence on accuracy in all other directions. Furthermore, the values observed in this clinical study demonstrate that computer-assisted surgical procedures can be recommended from a prosthetic point of view.

## Data Availability

The datasets used and/or analyzed during the current study are available from the corresponding author upon reasonable request.

## Abbreviations

3D: Three dimensional
CBCT: Cone beam computed tomography
STL: Standard Tessellation Language
